# Supplementary material for: Algorithmic Probability-Guided Machine Learning on Non-Differentiable Spaces
Source: Front Artif Intell. 2021 Jan 25;3:567356. doi: 10.3389/frai.2020.567356 (PMC7944352; doi:10.3389/frai.2020.567356)
Supplement: Supplementary file 1 [file datasheet1.pdf]

10 Appendix

10.1 Joint and Mutual BDM

In classical information theory ([34, 12]) we can think of *mutual entropy* as the information contained over two or more events occurring concurrently, and of *joint entropy* over the two communication channels or events as the average uncertainty contained over all possible combinations of events. For algorithmic information theory, the first concept can be understood as the "*amount of information within an object that is explained by another*" and the second concept can be interpreted as the "*amount of information contained within two or more objects*".

In contrast to classical information theory, we started by defining conditional BDM. Therefore we think that the best way to define joint BDM is from the *chain rule*.

Definition 10. The *joint BDM of X and Y with respect to* $\{\alpha_i\}$ is defined as

$$JointBDM(X,Y) = BDM(Y|X) + BDM(X).$$

Following the same path, we could define *mutual BDM* thus:

Definition 11. The *mutual BDM of X and Y with respect to* $\{\alpha_i\}$ is defined as

$$MutualBDM(X,Y) = BDM(X) - BDM(X|Y).$$

10.1.1 The Relationship Between Conditional, Joint and Mutual Information

The results shown in this section are evidence that our Def. for conditional BDM is *well behaved*, as it is analogous to important properties for conditional, joint and mutual entropy.

Proposition 12. *If $X = Y$ then $BDM(X|Y) = 0$.*

Proof. is a direct consequence of the Def. 5. □

It is important to note that $BDM(X|Y) = 0$ does not imply that $X = Y$. However, it does imply that $Adj(X) = Adj(Y)$. This is a consequence of the fact that BDM does not measure the information encoded in the position of the subtensors.

Proposition 13. $BDM(X) \geq BDM(X|Y)$.

Proof. As we consider subsets of $Adj(X)$, it is a direct consequence of the Def. 5. □

Proposition 14. *If X and Y are independent with respect to the partition $\{\alpha_i\}$, this is equivalent to $Adj(X) \cap Adj(Y) = \emptyset$, then $BDM(X|Y) = BDM(X)$.*

Proof. It is a direct consequence of the Def. 5, given that we have it that $Adj(X) - Adj(Y) = Adj(X)$. □

Proposition 15. $MutualBDM(X,Y) = MutualBDM(Y,X)$.

Proof. First, consider the equation

$$\begin{aligned} MutualBDM(X,Y) =& BDM(X) - BDM(X|Y) \\ =& \sum_{(r_i,n_i)\in Adj(X)} CTM(r_i) + \log(n_i) \\ &- \sum_{(r_i,n_i)\in Adj(X)-Adj(Y)} (CTM(r_i) + \log(n_i)) \\ &- \sum_{Adj(X)\cap Adj(Y)} f(n_k^x, n_k^y). \end{aligned}$$

While on the other hand we have it that

$$\begin{aligned} MutualBDM(Y,X) =& BDM(Y) - BDM(Y|X) \\ =& \sum_{(r_j,n_j)\in Adj(Y)} CTM(r_j) + \log(n_j) \\ &- \sum_{(r_j,n_j)\in Adj(Y)-Adj(X)} (CTM(r_j) + \log(n_j)) \\ &- \sum_{Adj(Y)\cap Adj(X)} f(n_k^y, n_k^x). \end{aligned}$$

Notice that in both equations we have the sum over all the pairs that are in both sets, $Adj(X)$ and $Adj(Y)$, with the difference being in the terms corresponding to the *multiplicity*. Now we have to consider two cases. If $n_i^x = n_i^y$ we have the equality. Otherwise, in the first equation we have terms of the form $\log(n_j^x) - f(n_k^x, n_k^y)$, which, by Def. of f, is 0; analogously for the second equation. Therefore, we have the equality. □

Proposition 16. *$MutualBDM(X,Y) = BDM(X) + BDM(Y) - JointBDM(X,Y)$.*

Proof.

$$\begin{aligned} MutualBDM(X,Y) &= MutualBDM(Y,X) \\ &= BDM(Y) - BDM(Y|X) \\ &= BDM(Y) + BDM(X) - (BDM(Y|X) + BDM(X)) \\ &= BDM(X) + BDM(Y) - JointBDM(X,Y) \end{aligned}$$

□

10.2 Coarseness and Relationship With Entropy

As mentioned in the previous section, the goal behind the Def. of coarse conditional BDM, $BDM(X|Y)$, is to measure the amount of information contained in X not present in Y. Ideally, this is measured by the conditional algorithmic information $K(X|Y)$. The Def. 5 includes the adjective *coarse* given that, as we will show in this section, its behaviour is closer to Shannon's entropy H than the algorithmic information measure K, relying heavily on the entropy-like behaviour of BDM.

The conditional algorithmic information content function K is an incomputable function. Therefore it represents a theoretical ideal that cannot be reached in practice. By construction, *coarse* conditional BDM is an approximation to this measure. However it differs in not taking into account two information sources: the information content shared between base blocks and the position of each block.

As an example of the first limitation, consider the string $101010\ldots10$ and its *negation* $010101\ldots01$. Intuitively, we know that both strings are algorithmically close, but for a partition strategy that divides the string into substrings of size 2 with no overlapping, the *Adj* sets $\{(\{10\},n)\}$ and $\{(\{01\},n)\}$ are disjoint. Therefore conditional BDM assigns the maximum BDM value to the shared information content. Within this limitation, we argue that conditional BDM represents a better approximation to K in comparison to entropy, mainly because BDM uses the CTM approximation value for each block, rather than just its distribution, and the information content of its multiplicity, thus representing a more accurate approximation to the overall algorithmic information content of the non-shared base blocks.

The second limitation can become a significant factor when the size of the base blocks is *small* when compared to that of the objects analysed, given that the positional information can become the dominant factor of the information content within an object. This is an issue shared with entropy

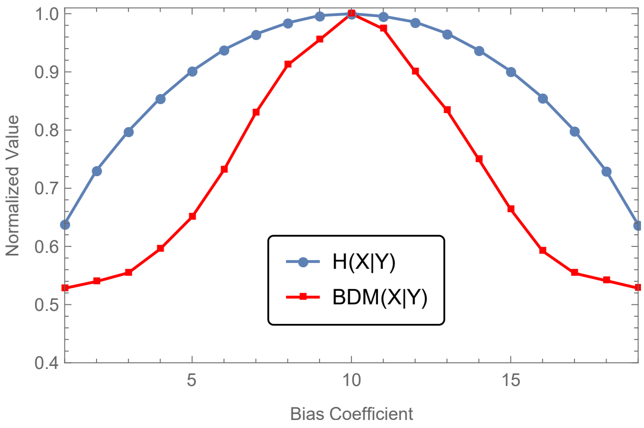

Figure 11: Each point represents the normalized average of the conditional BDM ($BDM(X|Y)$) and conditional entropy ($H(X|Y)$), respectively, corresponding to 5000 pairs of strings randomly chosen from a distribution where the expected number of 1s is the value shown on the x *axis* divided between the conditional BDM or conditional entropy of the first element of the pair and an unrelated randomly chosen binary string. All strings are of length 20. The partition strategy used for BDM is that of sets of size 1. From this plot we can see that conditional BDM manages to capture the statistical relationship of finite strings generated from the same distribution.

that conditional BDM inherits from the numerical challenges of CTM in BDM. However, conditional BDM has the added benefit that it is defined for finite tensors generated from different distributions by assuming the so-called *universal distribution* ([40]) (known to dominate any other approach) as the underlying distribution between the two '*events*'.

10.2.1 Empirical Comparison with Entropy

Owing to the origins of the BDM function, the asymptotic relationship between coarse conditional BDM and conditional entropy follows from the relationship between BDM and entropy ([49]). In this section we will focus on empirical evidence for this relationship, along with exploring the impact of the partition strategy for unidimensional objects. Further theoretical properties that establish the *well-behavedness* of conditional BDM are set forth in the Appendix in Section 10.1.

For this numerical experiment we generated a sample of 19,000 random binary strings of length 20 that are pairwise related, coming from one of 19 *biased* distributions where the expected number of 1s varies from 1 to 19. For each pair we computed the conditional BDM with partitions of size 1 and divided it by the conditional BDM of the first string with respect to a random string coming from an uniform distribution. To both, the divisor and the dividend, we added 1 to avoid divisions by zero. We repeated the experiment for conditional entropy. Both results where normalized by dividing the quotients obtained by the maximum value obtained for each distribution. In the plot 11 we show the average obtained for each biased distribution.

From the plot 11 we can see that as the underlying distribution associated with the strings is increasingly biased, the expected shared information content of two related strings is higher (conditional BDM is lower) when compared to the conditional BDM of two unrelated strings. This behaviour is congruent with what we expect and observe for conditional entropy. That the area under the normalized cube is smaller is expected, given that BDM is a finer-graded information content measure than entropy and is not perfectly symmetric, as BDM and CTM are computational approximations to an uncomputable function and are also inherently more sensitive to the fundamental limits of computable random number generators.

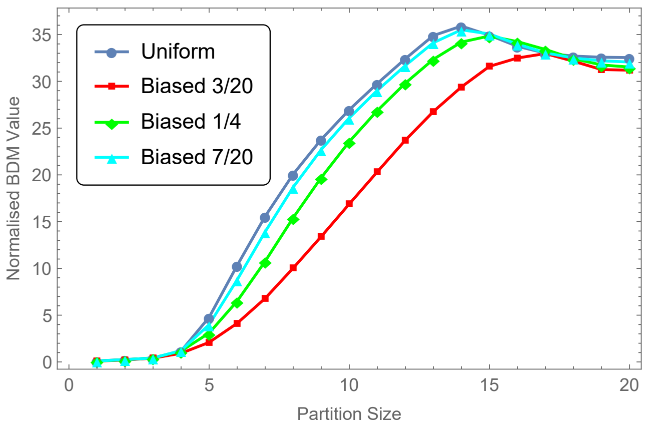

Figure 12: Each point represents the average of the conditional BDM from 30,000 pairs of binary strings of size 20 randomly generated from four different distributions: *uniform* (ten 1s expected), *biased 3/20* (three 1s expected), *biased 1/4* (five 1s expected) and *biased 7/20* (seven 1s expected). The *x axis* indicates the partition size used to compute the respective conditional BDM value, which was normalized by dividing it by the partition size.

10.3 The Impact of the Partition Strategy

As shown in previous results ([49]), BDM better approximates the universal measure $K(X)$ as the number of elements resulting from applying the partition strategy $\{\alpha_i\}$ to X. However, this is not the case for conditional BDM. Instead $BDM(X|Y)$ is a good approximation to $K(X|Y)$ when the $Adj(X)$ and $Adj(Y)$ share a high number of base tensors, and the probability of this occurring is lower in inverse proportion to the number of elements of the partition. For this reason we must point out that conditional BDM is dependent on the chosen partition strategy $\{\alpha_i\}$.

As a simple example, consider the binary string $X = 11110000$ and its inverse $Y = 00001111$. Since we have the CTM approximation for strings of size 8, the best BDM value for each string is found when $Adj(X) = \{(11110000, 1)\}$ and $Adj(Y) = \{(00001111, 1)\}$. However, given that the elements of the partitions are different, we have it that $BDM(11110000|00001111) = BDM(11110000) = 25.1899$,, even when intuitively we know that, algorithmic information-wise, they should be very close. However, conditional BDM is able to capture this with partitions of size 1 to 4 with no overlapping, assigning a value of 0 to $BDM(X|Y)$.

We conjecture that there is no general strategy for finding a *best partition strategy*. This is an issue shared with conditional block entropy, and just like the original BDM definition. At its worst, conditional BDM will behave like conditional entropy when comparable, while maintaining best cases close to the ideal of conditional algorithmic complexity. Thus the partition strategy can be considered an *hyperparameter* that can be empirically optimized from the available data.

We performed a numerical experiment to observe this behaviour by generating 2 400 000 random binary strings of size 20 with groups of 600,000 strings belonging to one of four different distributions: *uniform* (ten 1s expected), *biased 3/20* (three 1s expected), *biased 1/4* (five 1s expected) and *biased 7/20* (seven 1s expected). Then, we formed pairs of strings belonging to the same distribution and computed the conditional BDM using different partition sizes from 1 to 20, for a total of 30,000 pairs per data point, normalizing the result by dividing it by the partition size to avoid this factor being the dominant one. In the plot 12 we show the average obtained for each data point.

In figure 12 we can observe two main behaviours. The first is that as the partition size increases so does the conditional BDM value. This is because bigger partitions take into account more information from the position of each bit, and we do not expect randomly generated strings to share positional information. The drop observed after partitions of size 12 is the result of CTM values being available up to strings of size 12, the point where the program starts to rely on BDM for the computation.

Table 3: Accuracy for the first task

Classifier	Accuracy on `Test Set`	Accuracy on `Training Set`
Simple Networks		
1	60.11%	98.86%
2	57.30%	98.86
5	25.84%	32.95%
Fernandes	18.54%	50.56%
Algorithmic Class.	95.50%	96.02%

Table 4: Accuracy for the second task

Classifier	`Rules Test Set`	`Topology Test Set`
Logistic Regression	82.35%	20.75%
NN	92.50%	32.75%
Algorithmic Class.	91.35%	72.4%

Additionally the partition strategy ignores smaller partitions than the ones stated, thereby reducing the overall amount of information taken into account. The second is that not only is conditional BDM able to capture the discrepancies expected from the different distributions for partition sizes where there is no loss of statistical information (this being from size 1 to 10), but seems to improve on its ability to do so with larger partition sizes up to 10, therefore improving upon the results presented in Section 10.2.1.

It is important to note that an important reduction in accuracy for partitions of sizes larger than 10 was expected, given that the partition strategy used discarded substrings of smaller sizes than the ones stated. For instance, the partition of size 3 of the string 10111 is just {101}, thus losing information. Furthermore, for big partition sizes with respect to the string length, the statistical similarity vanishes, given that now each substring is considered a *different symbol of an alphabet*. Therefore, the abrupt change of behaviour observed beyond partitions of size 15 is expected and is the product of causation.

10.4 Experiments and models

$$M = \left\{ \begin{array}{llll} 704 & \rightarrow \{0,0,1,0,1,1,0,0,0,0,0,0\}, & 3572 & \rightarrow \{1,1,0,1,1,1,1,1,0,1,0,0\}, \\ 3067 & \rightarrow \{1,0,1,1,1,1,1,1,1,0,1,1\}, & 3184 & \rightarrow \{1,1,0,0,0,1,1,1,0,0,0,0\}, \\ 1939 & \rightarrow \{0,1,1,1,1,0,0,1,0,0,1,1\}, & 2386 & \rightarrow \{1,0,0,1,0,1,0,1,0,0,1,0\}, \\ 2896 & \rightarrow \{1,0,1,1,0,1,0,1,0,0,0,0\}, & 205 & \rightarrow \{0,0,0,0,1,1,0,0,1,1,0,1\}, \\ 828 & \rightarrow \{0,0,1,1,0,0,1,1,1,1,0,0\}, & 3935 & \rightarrow \{1,1,1,1,0,1,0,1,1,1,1,1\} \end{array} \right\} \quad (6)$$

10.5 Other result details

Table 5: Accuracy for the third task

Model	`Test Set`	`Training Set`
Neural Networks		
Naive	43%	100%
Convolutional	31.66%	97.66%
Boosted Trees	35%	64.33%
Algorithmic Classifiers		
BDM	70%	71%
Entropy	37.66%	46.33%
